# Identification of T- and B-Cell Subsets That Expand in the Central and Peripheral Lymphoid Organs during the Establishment of Nut Allergy in an Adjuvant-Free Mouse Model 

**DOI:** 10.1155/2013/509427

**Published:** 2013-04-11

**Authors:** Babu Gonipeta, David Duriancik, EunJung Kim, Elizabeth Gardner, Venu Gangur

**Affiliations:** ^1^Food Allergy and Immunology Laboratory, Michigan State University, East Lansing, MI 48823, USA; ^2^Department of Food Science and Human Nutrition, Michigan State University, East Lansing, MI 48823, USA; ^3^Division of Applied Life Science (BK 21 Program), Gyeongsang National University, Jinju, Republic of Korea

## Abstract

Nut allergies are potentially fatal and rarely outgrown for reasons that are not well understood. Phenotype of T- and B-cell subsets that expand during the early stages of nut allergy is largely unknown. Here we studied this problem using a novel mouse model of nut allergy. Mice were rendered hazelnut allergic by a transdermal sensitization/oral elicitation protocol. Using flow cytometry, the T- and B-cell phenotype in the bone marrow (BM), spleen, and the mesenteric lymph node (MLN) of allergic and control mice was analyzed. Nut allergic mice exhibited an expansion of CD4+ CD62L− T cells in BM and spleen; a similar trend was noted in the MLN. There was expansion of CD80+ B cells in BM and spleen and MLN and CD62L− cells in BM and spleen. Interestingly, among CD80+ B cells, significant proportion was CD73− particularly in the MLN. These data demonstrate that during the early establishment of hazelnut allergy there is (i) expansion of CD4+CD62L− T-cell subsets in both the BM and the periphery, (ii) expansion of CD80+ and CD62L− B-cell subsets in BM and the periphery, and (iii) a significant downregulation of CD73 on a subset of B cells in MLN.

## 1. Introduction

Food allergies such as tree nut allergies are potentially fatal group of immune-mediated disorders [1]. Recent studies demonstrate that both the prevalence and severity of food allergies are escalating for reasons that are not well understood at present [1, 2]. Tree nut allergies, along with peanut allergy, are the leading causes of food-induced systemic anaphylaxis in USA and European countries [3]. Furthermore, once individuals are sensitized, there is a very low potential for outgrowing tree nut allergies [4, 5]. Consequently, they are considered not only serious but also persistent health problems for rest of the life of sensitized subjects [4, 5].

The specific identity of T- and B-cell subsets that expand during the early stages of establishment of life-threatening nut allergies is largely unknown at present. Notably, most of the current knowledge about early expansion and establishment of immune memory cells comes from studies of infectious diseases [6–12]. Knowledge about identity of such immune cells is urgently needed for potential therapeutic targeting in nut allergies.

We have previously reported an adjuvant-free mouse model of tree nut allergy using hazelnut as a model tree nut [13]. This model employs a protocol combining transdermal sensitization followed by oral allergen challenge to elicit systemic anaphylaxis [13–15]. Furthermore, hazelnut allergy, once established, remains persistent even when the allergen is withdrawn for at least eight months in this model; and persistence of clinical sensitivity is associated with robust memory T-cell and memory B-cell responses [15]. Since this model does not use an external adjuvant, it offers a unique opportunity to determine the phenotype of T- and B-cell subsets that expand during the early stage establishment of nut allergy. Here we report the identification of the phenotype of T- and B-cell subsets that expand in the central and peripheral lymphoid organs during the establishment of nut allergy in this mouse.

## 2. Material and Methods

### 2.1. Reagents

Hazelnut protein extract (Greer Labs, Lenoir, NC, USA): protein content of these three protein extracts was measured by Lowry's method [16]. Normal saline was prepared in our lab (0.85% W/V NaCl solution): streptavidin alkaline phosphatase (Jackson ImmunoResearch, West Grove, PA, USA). The following fluorochrome-conjugated monoclonal antibodies were purchased from BD Biosciences (San Diego, CA, USA): CD3e (clone 145-2c11)-PerCp-Cy5.5, CD4(clone GK1.5)-APC-H7, CD8alpha(clone 53-6.7)-FITC, CD80(16-10A1)-FITC, and CD73(TY/23)-PE, while CD44(IM7)-PE, CD62L(MEL-14)-APC, and B220(RA3-6B2)-AlexaFluor700 were purchased from eBiosciences (San Diego, CA, USA).

### 2.2. Mice

All mice were purchased from Jackson Laboratories (Bar Harbor, ME, USA). All animals were females and used at 7-8 weeks of age. All procedures involving mice were in accordance with Michigan State University Animal Use Policies. Mice were acclimated for one week to their new environment before starting the experiment.

### 2.3. Transdermal Sensitization and Oral Elicitation of Systemic Anaphylaxis

Transdermal sensitization followed by oral allergen challenge protocols was performed using the method described previously [17, 18]. Sensitization was determined by measuring hazelnut-specific IgE antibody levels in the plasma using an ELISA-based method as described. Systemic anaphylaxis upon oral allergen challenge was quantified by rectal thermometry before and at 30 minutes after oral challenge using a digital temperature probe (Thermalert TH-5, Physitemp; NJ, USA; instrument specifications: resolution: 0.1°C; accuracy 0.1°C ±1 digit; operating range: 25–45°C). Animals were euthanized 1 hr after oral challenge and used for tissue collection.

### 2.4. Phenotype Analysis of T and B Cells by Flow Cytometry

Following euthanasia after 1 hour after oral challenge and collection of hypothermia data, bone marrow, spleen, and mesenteric lymph nodes were harvested, and single-cell suspensions were prepared using the standard protocols. One million cells were aliquoted for flow cytometry staining. Cells were blocked with antimouse CD16/32 (2.4G2 prepared in house) on ice for 10 minutes. Subsequently, cells were stained with either a memory T-cell master mix (CD3, CD4, CD8, CD44, and CD62L) or memory B-cell master mix (B220, CD80, CD73, CD44, and CD62L) of monoclonal antibody-fluorochrome conjugates. Each monoclonal antibody-fluorochrome conjugate was used at concentrations recommended by the manufacturer or previous antibody titration. Cells were incubated with the master mix for 30 minutes on ice in the dark, washed twice with FACS buffer, and run on a BD FACS Canto II flow cytometer. Compensation was established using BD Biosciences compensation beads.

Postacquisition flow cytometry analysis was performed using FlowJo software (Tree Star, Inc., Ashland, OR, USA). Live cells were gated based on forward scatter and side scatter for both T- and B-lymphocyte samples. T cells were gated as CD3+ on a histogram, CD4+ or CD8+ based on a dot plot, and subsequently a quadrant was drawn to separate CD44^hi^ and CD62L+ for each T-cell subset. Effector memory CD4 and CD8 T cells were defined as CD44^hi^/CD62L^neg^. Central memory CD4 and CD8 T cells were defined as CD44^hi^/CD62L [9, 19]. For B-cell analysis, histogram of B220^hi/+^ was drawn out of live cells, and a quadrant gate was used to distinguish CD62L+ and CD80+ cells. Expression of CD73 was analyzed by histogram from each quadrant. Increased expression of CD80, CD62L and CD73 is indicative of memory B-cell phenotype, and expression of CD73 may indicate isotype switched B cells [20, 21].

### 2.5. Statistical Analysis

ANOVA and Student's unpaired tests with Welch correction were used to evaluate significance using a software program (GraphPad software, San Diego, CA, USA). The statistical significance level was set at 0.05.

## 3. Results

### 3.1. Hazelnut Allergic Mice Exhibit a Significant Expansion of CD4+ CD62L– T Cells in the Bone Marrow and Spleen

**Figure 1:**
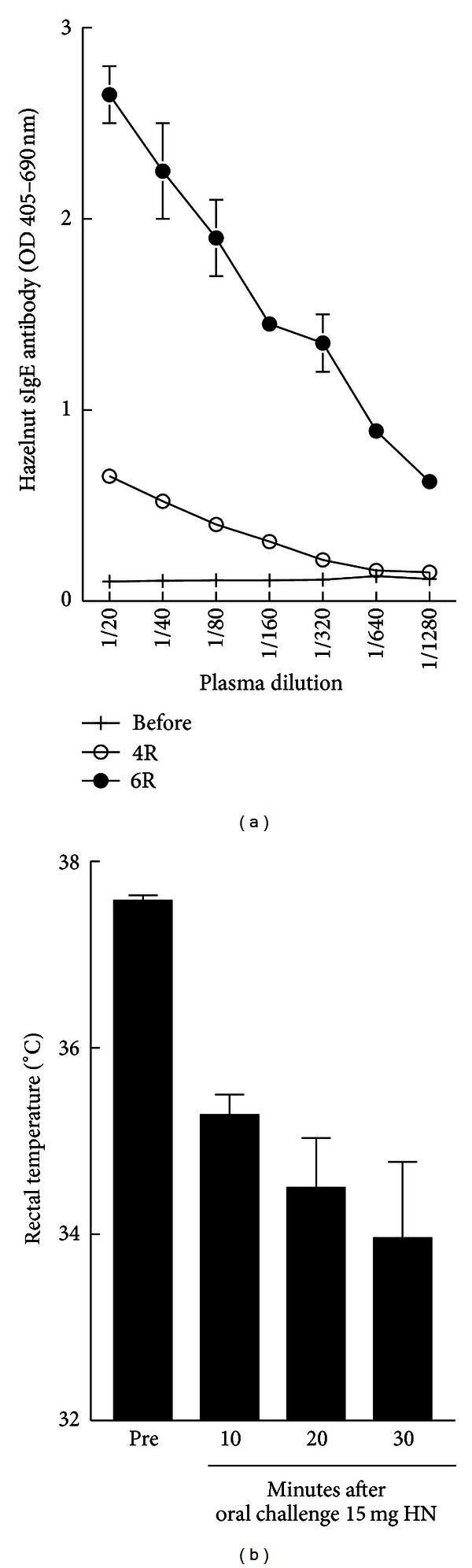
((a) and (b)) Transdermal sensitization followed by oral allergen challenge mouse model of hazelnut allergy. Groups of BALB/c (*n* = 5 to 7 per group) were sensitized with hazelnut (HN) protein (0.5 mg/mouse) via transdermal exposure as described in the method. Sensitization to hazelnut was confirmed by analysis of hazelnut-specific IgE antibody elevation in the serum upon exposure (a). Clinical reactions were evaluated by oral allergen challenge for up to 1 hr. Systemic anaphylaxis quantified by hypothermia responses is shown in (b).

Groups of mice were rendered hazelnut allergic using the transdermal sensitization followed by oral elicitation of systemic anaphylaxis to hazelnut that we have described before. The induction of hazelnut-specific IgE antibody response upon transdermal exposure is shown (Figure 1(a)). Systemic anaphylaxis to oral allergen challenge was confirmed by hypothermia responses (Figure 1(b)).

**Figure 2:**
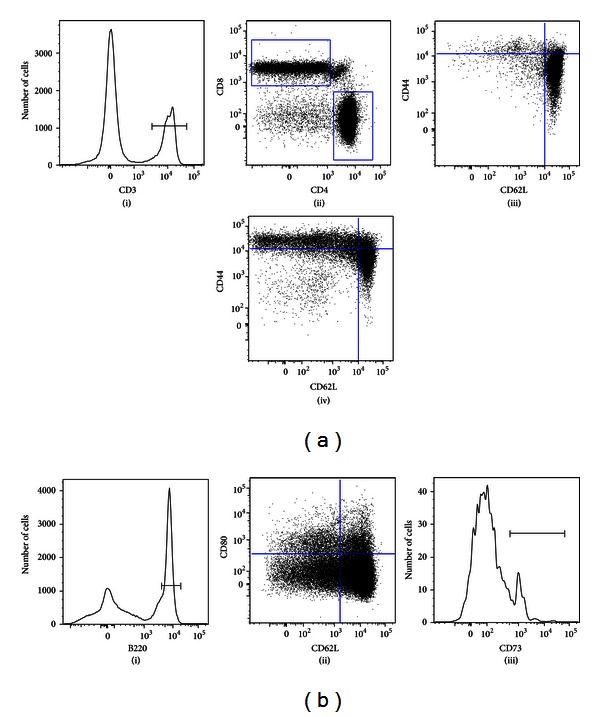
Gating strategy used to identify T- and B-lymphocyte subsets. Shown is one representative spleen sample. (a) Gating of memory T cells. Based on forward scatter, and side scatter live cells were gated and a histogram of CD3 (a(i)) was used to gate CD3+ cells. Subsequently, CD3+ cells were separated into CD4+ and CD8+ cells based on dot plot (a(ii)). Central memory T cells were gated as CD62L^hi^/CD44^hi^ and effector memory T cells were gated as CD62L^neg^/CD44^hi^ out of both CD4+ (a(iv)) and CD8+ T cells (a(iii)). (b) Gating of memory B cells. Based on forward scatter and side scatter, live cells were gated and a histogram of B220 (b(i)) was used to gate B220+ cells. In the bone marrow, only B220^hi^ cells were gated (data not shown). Subsequently, B cells were gated in the dot plot based on CD62L and CD80 expression (b(ii)). The expression of CD73 was assessed by histogram (b(iii)); only CD80+/CD62L^neg^ quadrant is shown as a representative example.

**Figure 3:**
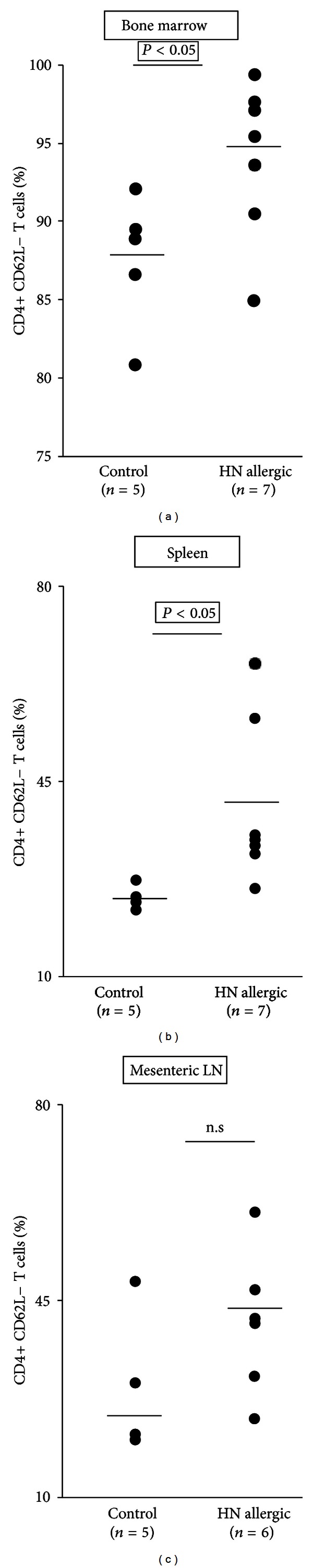
((a)–(c)) Analysis of central and peripheral phenotype of T lymphocytes from hazelnut allergic and nonallergic control mice. Groups of BALB/c (*n* = 5 to 7 per group) were sensitized with hazelnut protein (0.5 mg/mouse) via transdermal exposure and then confirmed for allergy by oral challenge. After 1 hour, bone marrow (a), spleen (b), and mesenteric lymph nodes (MLN) (c) were harvested and single-cell suspension prepared. Cells were stained with antibodies against CD3, CD4, and CD62L markers and analyzed by flow cytometry. Differences between allergic and nonallergic mice data were compared by Student's *t*-test for significance (*P* < 0.05).

The gating strategy we used, the phenotype of expanding T- and B-cell subsets, in the central and peripheral lymphoid organs is shown in Figures 2(a) and 2(b). We examined the subsets of CD4 cells for naïve versus memory phenotype using high CD62L expression as a marker of naïve cells and low CD62L expression as a marker of memory phenotype. As evident, there was consistent expansion of CD4+ CD62L− T cells in bone marrow and spleen (Figures 3(a)–3(c)). Although there was a similar trend in the MLN, it was not statistically significant.

### 3.2. Hazelnut Allergic Mice Exhibit a Modest Increase in CD4+ CD44+ T Cells in the Central and the Peripheral Immune Compartments

We also examined the subsets of CD4 cells for naïve versus memory phenotype using high CD44 expression as a marker of memory cells and low CD44 expression as a marker of naive phenotype. As evident, there was a modest increase in the number of CD4+ CD44+ T cells in bone marrow and spleen, but this was not statistically significant (Table 1).

### 3.3. Hazelnut Allergic Mice Exhibit a Modest Increase in CD80+ and CD62L− B Cells in Both Central and Peripheral Lymphoid Organs

Then we examined the B cells for naïve versus memory phenotype using antibodies to CD80 and CD62L as markers. As evident, there was a modest increase in the proportion of CD80+ B cells in the BM, spleen, and MLN. A similar trend was noticed for CD62L− B cells in BM and spleen but not in MLN (Table 2).

### 3.4. Hazelnut Allergic Mice Exhibit a Significant Downregulation of CD73 on CD80+ B Cells in the MLN

**Figure 4:**
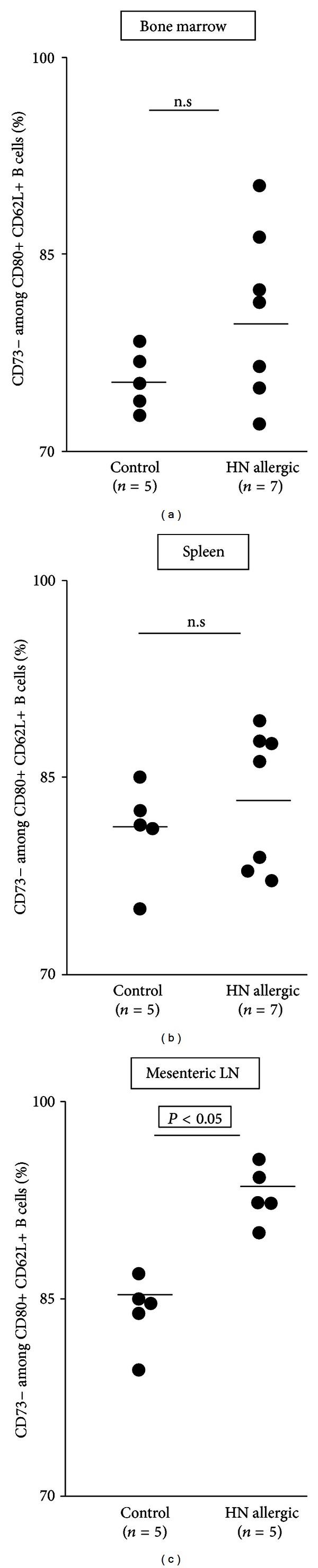
((a)–(c)) Analysis of central and peripheral phenotype of B lymphocytes from hazelnut allergic and nonallergic control mice. Groups of BALB/c (*n* = 5 to 7 per group) were sensitized with hazelnut protein and then confirmed for allergy by oral challenge. After 1 hour, bone marrow (a), spleen (b), and mesenteric lymph nodes (MLN) (c) were harvested and single-cell suspension prepared. Cells were stained with antibodies against surface markers CD80, CD62L, and CD73 and analyzed by flow cytometry. Differences between allergic and nonallergic mice data were compared by Student's *t*-test for significance (*P* < 0.05).

We surprisingly found a significant reduction in CD73 expressing cells among B cells with both CD80+ CD62L+ and CD80+ CD62L− phenotypes in the mesenteric lymph nodes but in bone marrow or the spleen (Figures 4(a)–4(c)).

## 4. Discussion

This study was undertaken to identify the phenotype of T- and B-cell subsets that expand during the early stages of establishment of hazelnut allergy in an adjuvant-free mouse model that we have described and characterized before [13–15, 22]. We studied the immune cell expansion in the bone marrow as a central lymphoid organ, spleen as a systemic peripheral lymphoid organ, and the mesenteric lymph node as representative draining lymphoid organ of the gut. There are three novel and important findings from this study: (i) we report a significant expansion of CD4+ CD62L− T cells in both the BM and the periphery; (ii) an increase in CD80+ and CD62L− B cells in BM and the periphery; (iii) a significant downregulation of CD73 expression on a subset of B cells particularly in the MLN.

We have previously reported that hazelnut allergy once established in model remains persistent for up to 8 months despite withdrawing allergen exposure [15]. However, the specific phenotype of immune subsets that expand at early stages of the disease establishment in the allergic mice was unknown. As a first necessary step towards the long-term goal of identifying the phenotype of persistent T- and B-cell subsets in this model, here we characterized the phenotype of immune cells that expand early on in this model. We are not aware of previous studies examining this in hazelnut allergy or other models of nut food allergy. 

It has long been held that memory B cells that expand during immune responses during the early stages reside in the BM and memory T cells reside in the lymph nodes [23–25]. However, recent studies clearly demonstrate that nearly 80% of antigen-specific memory T cells reside in the BM in mice upon infection [9]. Our data demonstrate for the first time that hazelnut causes significant expansion of CD4+ CD62L− T cells in BM as well as in the periphery. In addition there was a modest elevation of such B cells in BM or the spleen that was not statistically significant.

One previous study examined memory T-cell phenotype in a mouse model of airways allergy/asthma [26]. They reported expansion of CD62L− memory T cells in the lungs of mice with chronic asthma. However, they did not study bone marrow. It is noteworthy that our data using an adjuvant-free mouse model also demonstrates that CD4+ CD62L− T cells are induced in both the spleen and bone marrow in response to hazelnut exposure. Clearly, more studies are needed to test whether they are long-lasting memory cells and to determine whether long-term persistence of hazelnut allergy in this model is associated with long-lived such subsets in bone marrow or spleen or both. 

The surface marker, CD73, is a glycosyl-phosphatidylinositol- (GPI-)anchored signaling molecule expressed on both T and B cells [20, 27]. Other studies have identified CD73 as an important marker of B-cell memory subsets [21]. Because of this significance, we examined CD73 and found that a significant proportion of CD80+ B cells downregulates CD73 expression in allergic mice. This suggests that this unusual subset of cells might represent a subset of quiescent B cells that we hypothesize as a potential subset of memory B cells.

Based on previous reports in the literature, one can use CD62L as a marker to distinguish naïve versus memory T cells and CD80, CD62L, and CD73 as markers of memory B cell subset phenotype [21]. We, however, acknowledge the fact that memory cells are known to be and expected to be heterogeneous, and choosing only a few selected markers is a limitation of our study. Also cell analyses were done in mice after they were orally challenged with the allergen to induce shock. Therefore, shock-induced redistribution of cells (if at all occurred in this short time of 1 hour, which is very unlikely) also possibly contributed to the observations in this study.

These data demonstrate for the first time that during the early stages of establishment of hazelnut allergy there is (i) expansion of CD4+ CD62L− T cell subset in both the BM and the periphery, (ii) expansion of CD80+ and CD62L− B-cell subset in BM and the periphery, and (iii) a significant downregulation of CD73 on a subset of B cells particularly in MLN. 

## Figures and Tables

**Table 1:** Analysis of CD4+ CD44+ memory T cells in hazelnut allergic and nonallergic control mice.

Group	Percentage of CD4+ CD44+ T cells		
	Bone marrow	Spleen	Mesenteric lymph node
Control mice (*n* = 5)	21.18 ± 2.1	15.19 ± 0.4	7.31 ± 0.5
Hazelnut allergic mice (*n* = 6-7)	24.09 ± 2.3	18.92 ± 2.4	5.51 ± 1.1

**Table 2:** Analysis of B-cell phenotype in hazelnut allergic and nonallergic control mice.

Group	Bone marrow				Spleen				Mesenteric lymph node			
	CD80+	CD80−	CD62L+	CD62L−	CD80+	CD80−	CD62L+	CD62L−	CD80+	CD80−	CD62L+	CD62L−
Control mice (*n* = 5)	25.1 ± 1	74.9 ± 2.4	55.8 ± 1.1	44.2 ± 1.1	9.3 ± 0.2*	90.6 ± 0.2	74.2 ± 1.1	25.7 ± 1.1	12.3 ± 1.1	87.6 ± 1.1	57.4 ± 4.7	42.5 ± 6.7
HN allergic mice (*n* = 5–7)	31.1 ± 7.4	68.9 ± 7.4	49.0 ± 8	50.8 ± 8	10.5 ± 0.3	89.4 ± 0.3	67.1 ± 6.3	32.8 ± 6.3	16.6 ± 2.3	83.3 ± 2.3	56.9 ± 7.1	43 ± 7.1

*Significantly different, *P* < 0.05.
